# Display of wasp venom allergens on the cell surface of *Saccharomyces cerevisiae*

**DOI:** 10.1186/1475-2859-9-74

**Published:** 2010-09-24

**Authors:** Irina Borodina, Bettina M Jensen, Ib Søndergaard, Lars K Poulsen

**Affiliations:** 1 Center for Microbial Biotechnology, Institute of Systems Biology, Technical University of Denmark, Søltofts Plads 224, 2800 Kgs. Lyngby, Denmark; 2 Dermato-Allergological Dept. K, CUH-Gentofte, Rigshospitalet Dept 7551, Blegdamsvej 9, 2100 København Ø, Denmark

## Abstract

**Background:**

Yeast surface display is a technique, where the proteins of interest are expressed as fusions with yeast surface proteins and thus remain attached to the yeast cell wall after expression. Our purpose was to study whether allergens expressed on the cell surface of baker's yeast *Saccharomyces cerevisiae *preserve their native allergenic properties and whether the yeast native surface glycoproteins interfere with IgE binding. We chose to use the major allergens from the common wasp *Vespula vulgaris *venom: phospholipase A1, hyaluronidase and antigen 5 as the model.

**Results:**

The proteins were expressed on the surface as fusions with a-agglutinin complex protein AGA2. The expression was confirmed by fluorescent cytometry (FACS) after staining the cells with antibody against a C-tag attached to the C-terminal end of the allergens. Phospholipase A1 and hyaluronidase retained their enzymatic activities. Phospholipase A1 severely inhibited the growth of the yeast cells. Antigen 5 - expressing yeast cells bound IgE antibodies from wasp venom allergic patient sera but not from control sera as demonstrated by FACS. Moreover, antigen 5 - expressing yeast cells were capable of mediating allergen-specific histamine release from human basophils.

**Conclusions:**

All the three major wasp venom allergens were expressed on the yeast surface. A high-level expression, which was observed only for antigen 5, was needed for detection of IgE binding by FACS and for induction of histamine release. The non-modified *S. cerevisiae *cells did not cause any unspecific reaction in FACS or histamine release assay despite the expression of high-mannose oligosaccharides.

In perspective the yeast surface display may be used for allergen discovery from cDNA libraries and possibly for sublingual immunotherapy as the cells can serve as good adjuvant and can be produced in large amounts at a low price.

## Background

Identification and characterization of allergenic compounds is essential for development of advanced component-resolved allergy diagnostics and treatment [1,2]. Single allergens can be identified either by resolving an allergenic extract into single proteins or by recombinantly expressing a library of allergenic genes in a host organism. In the later approach phage display in *E. coli *is commonly used [3,4]. However, *E. coli *is known to fail to express a number of eukaryotic proteins due to the lack of foldases and chaperones, which are important for the correct folding of proteins. Most allergens have conformational IgE epitopes, which might disappear if the protein is folded incorrectly. This can represent a limitation of a phage display. Yeast offers an alternative approach for display and selection of antigens and antibodies. Firstly, it provides a wider repertoire of correctly folded and glycosylated proteins, secondly, it allows a more convenient and faster screening of positive clones by fluorescent-activated cell sorting. Bowley *et al. *[5] compared phage and yeast display for their ability to express HIV-1 immune scFv cDNA library. The obtained clones were screened with the same selecting antigen (HIV-1 gp120). Yeast library was far superior to the phage display library selecting all the scFv identified by phage display and twice as many novel antibodies. In another study Wadle *et al. *[6] identified 33 novel breast cancer-related antigens using yeast surface display library, of these only four were found previously when using bacterial-based libraries. In addition to being useful for novel antigens discovery, the yeast display can be used in various other applications such as protein engineering, immunoassays, affinity purification and as vaccines [7]. Baker's yeast *Saccharomyces cerevisiae *has GRAS (generally regarded as safe) status, which simplifies its use in pharmaceutical applications. To the best of our knowledge, there are no previous literature reports of yeast surface display technology applied to allergens. A major obstacle in the application of yeast surface display for expression of allergens could be interference from the high-mannose oligosaccharides, which may either bind IgE or hinder IgE binding to the peptide-determinant. IgE antibodies directed towards carbohydrate epitopes (cross-reactive carbohydrate determinants, CCDs) are common in sera of patients allergic to insect venoms and plant allergens [8]. For example, the anti-CCD IgE were found in 28% of honey-bee venom allergic patients [9], 33% of grass-allergic patients [10], and 45-55% of carrot-celery allergic patients [11,12]. However, a number of studies have shown that the anti-CCD IgE are commonly directed towards core α1,3-fucose, which does not occur in yeast [8,13].

**Figure 1:**
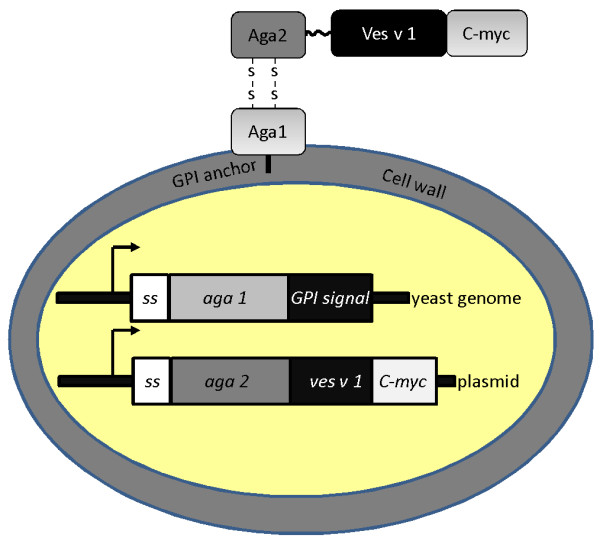
**The principle of surface display using a-agglutinin system**. The allergen of interest (here shown *ves v 1*) is cloned in-frame with *aga2 *gene and introduced into a yeast strain that can overexpress *aga1 *gene. The resulting proteins, Aga1 and Aga2-allergen fusion, get connected by disulfide bonds in the secretion process and are exported together on the surface of the cell where Aga1 remains attached to the cell wall by the glycosylphosphatidylinositol (GPI) anchor.

There are several systems that allow display on the yeast cells, based on a-agglutinin, α-agglutinin or flocculin proteins. A- and α-agglutinins mediate the cell-cell adhesion during the yeast cell mating and are located on the outmost surface of yeast cells. a-type mating cells express a-agglutinin, which consists of AGA1 subunit attached to the cell wall by glycosylphosphatidylinositol (GPI) anchor and of a small AGA2 subunit connected with AGA1 by disulfide bridges. The DNA sequence encoding protein of interest can be fused to the C-terminal of *aga2 *gene and transformed into an *aga1*-overexpressing *S. cerevisiae *strain, which will result in the protein being expressed as fusion with AGA2 on the outmost cell surface of the yeast cells (Figure 1).

We have chosen common wasp *Vespula vulgaris *venom allergens as the model for testing allergen expression on the surface of *S. cerevisiae*. The diagnosis and treatment of stinging insect allergies as wasp and honey bee have been used as an allergy model for several decades [14]. After the substitution of whole body extract therapy by venom extracts in 1979, the efficacy of treatment reached 95% for wasp venom allergy and 80% for honey bee venom allergy [15]. The composition of the vespid venoms has been extensively studied and the major allergic components have been identified [16]. On average there is 1.7-17 μg of protein in a vespid venom sac with 3.3% being phospholipase A1, 1.5% hyaluronidase, and 8.1% antigen 5, which all can cause allergic response in humans [17,18]. Phospholipase A1 is a 35 kDa non-glycosylated enzyme; antigen 5 is a 23 kDa non-glycosylated protein with so far unidentified function [19]. Hyaluronidase is a 45 kDa glycoprotein, in which insect-type glycosylation with α1,3-fucoses is believed to be responsible for cross-reactivity with sera of honey-bee sensitized patients [8,20]. Three allergens from the common wasp venom have been expressed recombinantly in *E. coli *[21-24] and insect cells [25,26] and antigen 5 was additionally expressed in yeast *Pichia pastoris *[23].

Here we describe expression and characterization including the biological activity of three major wasp venom allergens phospholipase A1, hyaluronidase and antigen 5 on the surface of *S. cerevisiae *cells.

## Results and discussion

### Cloning of allergen genes

The genes encoding major wasp allergens phospholipase A1 (Ves v 1.0101, further called Ves v 1), hyaluronidase (Ves v 2.0101, further called Ves v 2) and antigen 5 (Ves v 5.0101, further called Ves v 5) were amplified from the total RNA of common wasp *V. vulgaris *by RT-PCR. We also attempted amplifying the gene, encoding a hyaluronidase-like protein without enzymatic activity (Ves v 2.0201), which was found previously in *V. vulgaris *and *V. germanica *venoms [25], but only non-specific gene products of *ves v 2.0201 *were obtained. The genes were subsequently cloned into TOPO vector and sequenced.

There were a few differences from the gene sequences reported by King's group [21,27]. Phospholipase A1-encoding gene had six variations, five of which were single point mutations and one was a change of all the three nucleotides in a triplet. For hyaluronidase 4 nucleotide discrepancies from the previously published sequence were observed, all were silent point mutations; hence, the amino acid sequence remained as reported before. Hyaluronidase is known to be more conserved than phospholipase A1. So common wasp hyaluronidase has 92% identity with amino acid sequence of a homolog from white faced hornet, while phospholipase A1 has only 67% identity [21]. The sequence of antigen 5-encoding gene had six single nucleotide variations from King's sequence, four of which have been reported elsewhere (GenBank accession nr. AJ238849). It is important to note that neither of the variations was positioned in the predicted active site (UniProt http://www.uniprot.org) or affected cysteins, hence the disulfide bond formation should not be disturbed.

In general, the variations in the gene sequences can be explained firstly by different geographical locations as the wasps in King's work were from Vespa Labs (PA, USA), while our wasps were from Denmark in Europe. Secondly, it can come from polymorphism, which has been described even for genes within the same vespids' population. Thus Hoffman sequenced Ves m 1 protein from *V. maculifrons *and Dol m 1.02 from white faced hornet *Dolichovespula maculate *and found 3 amino acids variant positions in Ves m 1 and 2 variants positions in Dol m 1.02 [28]. Soldatova, Kochoumian and King [29] described 4 natural variants of *dol m 1.01 *mRNA sequence.

### Expression of allergens on the surface of yeast

**Figure 2:**
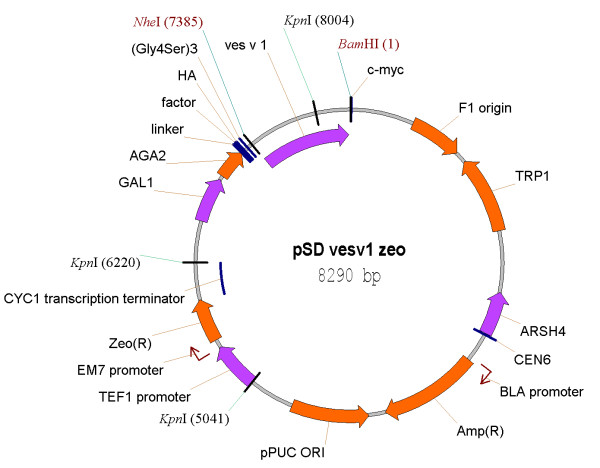
**Vector map of a surface display vector with zeocin resistance**. The original vector pCTCON2 [36] was modified by addition of zeocin resistance cassette, containing yeast TEF1 promoter, prokaryotic EM7 promoter, zeocin resistance gene *ble *from *Streptoalloteichus hindustanus *and transcription terminator CYC1. The allergen genes were cloned flush downstream the flexible peptide linker (Gly_4_Ser)_3_, which was connected to *aga2 *gene under control of galactose-inducible promoter GAL1. The allergen genes were also cloned in-frame with C-terminal myc tag. The vector additionally contained ampicillin resistance marker Amp(R) for selection in *E. coli*, tryptophan selection marker TRP1 for selection in *S. cerevisiae*, pUC origin of replication for *E. coli*, and CEN6/ARSH4 origin for vector replication in *S. cerevisiae*.

**Figure 3:**
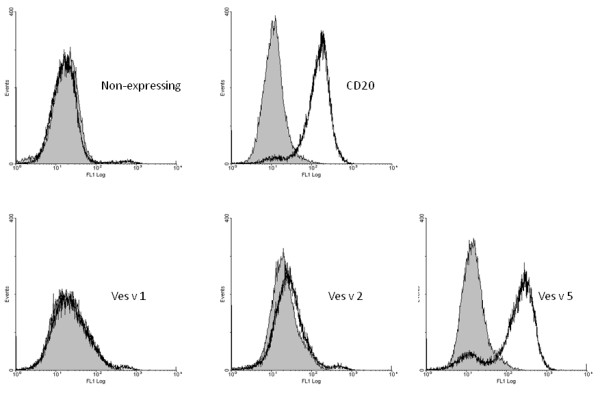
**Fluorescent cytometry of yeast cells expressing reference protein (CD20) and wasp venom allergens**. The cells induced on galactose under selective pressure of tryptophan and zeocin to maintain expression plasmid were labeled with AlexaFluor488-conjugated anti-C-myc antibody and analyzed by FACS. The grey background shows non-stained cells.

The genes were PCR-amplified with overhangs and directionally cloned into a-agglutinin expression vector to make an in-frame fusion with *aga2 *gene (Figure 1, 2). The plasmids were transformed into *S. cerevisiae *strain EBY100, which can overexpress another protein of the a-agglutinin system - AGA1. When the cells were grown on inducing medium, the AGA1 and AGA2-allergen fusion was expressed and exported on the cell surface as a complex held by disulfide bonds (Figure 1). A 51-amino acid long fragment of human CD20 protein was used as a non-allergen control. CD20 antigen is expressed on the surface of mature B-lymphocytes and to the best of our knowledge there are no reports on the presence of IgE antibodies to this autologous protein in human sera. The allergens also contained a 10 amino acid-long C-myc tag at the C-terminal. The expression of allergens was quantified by fluorescent cytometry after staining the expressing cells with anti-C-myc antibody conjugated with AlexaFluor488 (Table 1). The expression of antigen 5 was almost just as high as of the reference protein CD20; however there was very weak expression of phospholipase A1 and hyaluronidase. We speculate that these allergens might be toxic to the yeast cells because of their enzymatic activity. The a-agglutinin vector is a replicating vector, which is retained in the cells due to tryptophan selection. As tryptophan is an amino acid needed in very small amounts, the selection is not very strong and the vector can be lost by the cells. To circumvent the problem we introduced zeocin resistance marker into the vector and performed cultivation in the presence of zeocin antibiotic so that constant selective pressure was applied (Figure 3, Table 1). The expression level of CD20 and antigen 5 improved from when only tryptophan selection was used; now correspondingly 74.9 and 73.4% of the cells expressed the proteins against 43.7 and 32.8% before. Moreover increased mean ratio indicated higher amount of displayed protein per cell. The hyaluronidase and phospholipase A1 expressing cells had increased autofluorescence, which can be a sign of stress. About 5-4% of the cells expressed phospholipase A1 and hyaluronidase, though the amount of allergens per expressing cell was much lower than for antigen 5 or CD20, as can be judged from the low mean ratios (Table 1). Besides improving the allergen expression introduction of zeocin selection also had a positive effect on selecting correct yeast transformants as the false positives were fully eliminated.

**Table 1:** Quantification of allergen expression on the surface by FACS analysis

	% of expressing cells		Geometrical mean ratio	
	
Protein	Tryptophan selection	Tryptophan and zeocin selection	Tryptophan selection	Tryptophan and zeocin selection
None	2.4	-	1.0	-
CD20	43.7 ± 1.0	74.9 ± 1.3	3.1 ± 0.2	11.5 ± 0.3
Phospholipase A1 (Ves v 1)	3.3	5.6	1.2	1.1
Hyaluronidase (Ves v 2)	5.1	5.3	1.3	1.5
Antigen 5 (Ves v 5)	32.8	73.4	2.2	9.3

The cells were stained with anti-C-myc antibody conjugated with AlexaFluor488 and analyzed by fluorescent flow cytometry. The intensity of the green fluorescent signal was measured. For calculation of percentage of expressing cells and geometrical mean ratio the labeled cells were compared with non-labeled cells. The labeling of CD-20-expressing cells was performed in triplicates and the average and standard deviation are shown.

**Figure 4:**
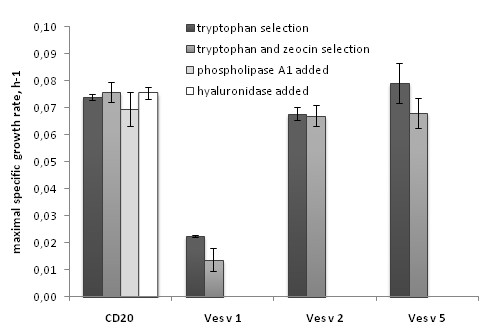
**Maximal specific growth rates of yeast cells expressing reference protein (CD20) and wasp venom allergens**. Maximal specific growth rates of cells grown on galactose under tryptophan selection (first columns) or combined tryptophan and zeocin selection (second columns) were measured in triplicates. The error bars show standard deviation. Two extra columns demonstrate the effect of extracellular addition of phospholipase A1 or hyaluronidase on the growth rate of CD20-expressing cells.

In order to evaluate toxicity of the allergens to the expressing yeast cells we performed growth rate experiments, which showed that the phospholipase A1 expression decreased the maximal specific growth rate by 70% and 82% when cells were grown without or with zeocin selection correspondingly (Figure 4). In contrast, addition of phospholipase A1 (Lecitase^® ^Ultra) directly into the growth medium with CD20-expressing yeast in the amount of 3.3 u/ml (which is about an order of magnitude higher that the expressing cells can accumulate at the given conditions) did not have a reverse effect on the growth rate. Most likely the cells are protected from the extracellular phospholipase by their cell wall and therefore are not influenced by its presence in the medium, while the phospholipase which is produced inside the cells damages them during synthesis and secretion. The hyaluronidase expression caused a statistically significant decrease in the maximal specific growth rate (p < 0.05), though much less pronounced than the phospholipase A1: 8% and 11% for cells grown with tryptophan or with tryptophan-zeocin selection correspondingly. The external addition of *Streptomyces hyalurolyticus *hyaluronidase at the concentration of 17 u/ml did not affect the growth (the enzyme activity exceeds the recombinant hyaluronidase activity that the cells can accumulate by an order of magnitude).

Expression of phospholipase A1 and hyaluronidase might also be limited by the capacity of yeast endoplasmic reticulum to fold these heterologous proteins, which would result in unfolded protein response, characterized by loss of protein expression and various stress reactions [30]. Lower cultivation temperatures have been shown to reduce the unfolded protein response and can be attempted in future studies to improve the cell surface expression of difficult proteins [31].

### Enzymatic activity of the surface-bound allergens

We tested the enzymatic activity of the yeast cells expressing phospholipase A1 and hyaluronidase. The enzymatic activity of phospholipase A1 was measured to 0.08 and 2.6 units/10^9 ^cells when grown with only tryptophan or tryptophan/zeocin selection correspondingly. Hence the addition of zeocin selection improved the expression of phospholipase A1 by an order of magnitude. For the hyaluronidase an improvement was also found. Here the numbers were 31 and 90 units/10^9 ^cells, which is a 3-fold increase.

### Binding IgE from patient serum by FACS

**Figure 5:**
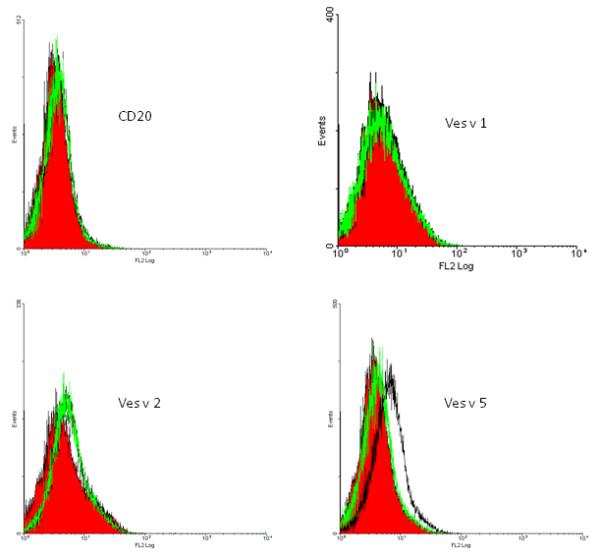
**Fluorescent cytometry analysis of cells binding to serum IgE**. Binding of yeast cells to the IgE from sera. Red background shows non-labeled cells, black line shows cells labeled with positive serumpool and the green line shows cells labeled with control serumpool.

The ability of the surface-expressed allergens to bind human IgE antibodies was tested by incubating the yeast cells with serum, staining with anti-IgE biotinylated antibody and streptavidin-phycoerythrin and analyzing the cells by flow cytometry (Figure 5). A serumpool from five wasp venom allergic patients was used as positive control and a serumpool from healthy individuals was used as negative control. Preceding FACS analysis the pool of wasp venom allergic sera was tested by western blot with venom extract to confirm that IgE against all the three allergens were present (data not shown).

**Figure 6:**
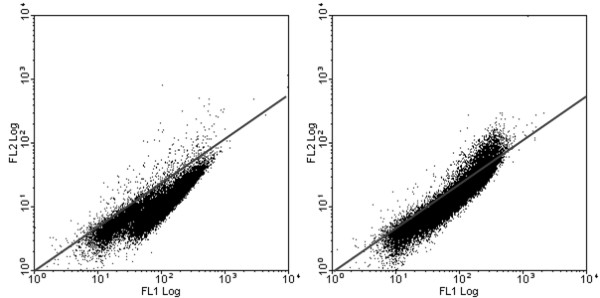
**Correlation between allergenic protein expression and binding to serum IgE**. Double staining of cells expressing CD20 (left) or Ves v 5 (right) with patient sera IgE and anti-C-myc antibody. The anti-C-myc binding is measured in FL1 and the IgE binding in FL2.

The yeast cells without surface protein or expressing the reference CD20 protein did not bind IgE antibodies from either serumpool. Antigen 5-expressing cells bound IgE from the wasp venom allergic serumpool, but not from the control serumpool. Hyaluronidase-expressing cells bound very limited IgE but from both serumpools indicating an unspecific IgE effect. The binding of IgE to phospholipase A1 could not be detected presumably because of the low expression level. Double staining with anti-C-myc antibody and IgE confirmed that it is the cell population that binds anti-C-myc and hence has the allergen protein expressed that also gives a signal for IgE binding (Figure 6).

### Histamine release assay

**Figure 7:**
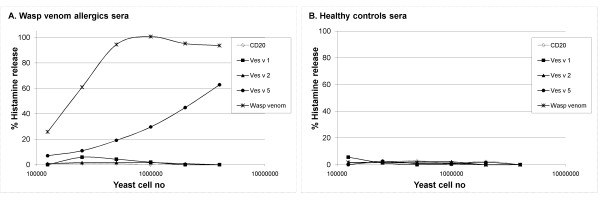
**Basophil histamine release**. Basophils sensitized with serum from wasp venom allergics (A) or healthy controls (B) were challenged with wasp venom and yeast cells expressing CD20, Ves v 1, Ves v 2 or Ves v 5 (from surface display vectors with zeocin-resistance markers). The results represent one experiment out of four. The wasp venom is diluted 3-fold in each step from the highest concentration of 222 ng venom extract per sample.

The allergen-expressing cells were tested for their ability to mediate a histamine release from blood basophils sensitized with IgE from wasp venom allergics or healthy controls. Basophils were challenged with wasp venom to illustrate the wasp venom specific response and as seen in Figure 7, only the basophils sensitized with serum from wasp venom allergics released histamine.

Comparable to the IgE-binding analysis, the CD20-expressing cells did not mediate a histamine release. Hyaluronidase did not mediate a histamine release supporting that the unspecific IgE binding seen with FACS is not biologically relevant. In 1 out of 4 experiments phospholipase A1 mediated an unspecific histamine release since both wasp venom sensitive and control basophils were activated (data not shown). Antigen 5 however did activate the wasp venom sensitive basophils and a dose response curve could be generated as seen in Figure 7A. The potency of 10^6 ^antigen 5 - expressing cells correspond to about 1 ng of wasp venom extract. Antigen 5 did not activate basophils sensitized with control serum.

## Conclusions

The aim of the study was to test whether allergenic proteins retained their IgE-binding activity when expressed on the surface of yeast cells. Moreover, we aimed at studying whether allergen displaying yeast cells expressed allergenic activity in a biological model of the allergic reaction: the basophil histamine release test. We chose wasp venom allergens as the model allergens, which proved to be a difficult case due to the cellular toxicity of one allergen (phospholipase A1) and low expression of another allergen (hyaluronidase) for unknown reasons. However, some levels of expression of both allergens were obtained and corresponding enzymatic activities were detected, suggesting that the proteins obtained a certain degree of their native confirmation. There have been no reports so far on recombinant expression of these two proteins in a yeast host. The last allergen that we tested, antigen 5 was well expressed on the surface of yeast cells; it retained IgE binding and could induce allergen-specific histamine release. The non-modified yeast showed neither IgE binding in the FACS assay nor activity in the histamine release assay.

The procedure for allergen expression on the surface is fast and the cells can be tested directly for IgE binding by FACS and histamine release without the need for recombinant protein purification. As an eukaryotic host, yeast is more likely to fold eukaryotic proteins more efficiently and to form disulfide bonds correctly, which eliminates the need of refolding the proteins as after expression in *E. coli *[32,33]. The proteins made by yeast are also N-glycosylated [34], which is sometimes important for proper folding as in the case of hyaluronidase [35]. It should be mentioned that the glycosylation is of high-mannose type and its interaction with human immune system is largely unknown [8]. We, however, did not observe IgE binding to yeast cells that could be related to carbohydrate-determined IgE reactivity. The immunogenic reactivity of antigen 5 should not be related to CCD reactivity, because this protein is not N-glycosylated neither in its native form in the venom nor when expressed recombinantly in yeast *P. pastoris *[23].

The expression of allergens on the surface of yeast cells presents new opportunities as identification of novel allergens in yeast display cDNA libraries and immunotherapy with yeast cells.

## Methods

### Patients

5 patients were chosen based on their serum IgE reactivity with venom extract in ImmunoCap assay (Phadia), all CAP class 4 or above. The equal volumes of sera were pooled and used as the positive sera. The negative control sera pool consisted of 3 sera from non-allergic individuals as confirmed by negative histamine release assay with wasp venom. The usage of patients' sera in the current study was approved by the local ethical committee.

### Chemicals

The chemicals were purchased from Sigma-Aldrich and BD Biosciences. Zeocin™ was purchased from Invitrogen. Anti-C-myc monoclonal mouse IgG antibody, conjugated to horse radish peroxidase, was purchased from Invitrogen. Mouse monoclonal anti-C-myc antibody (clone 9E10) conjugated with AlexaFluor^® ^488 was from AbD Serotec. Biotin-conjugated mouse anti-human IgE antibody (clone G7-26) and streptavidin-phycoerythrin (PE) conjugate (SAv-PE) were from BD Biosciences. Hyaluronic acid potassium salt from human umbilical cord and hyaluronidase from *Streptomyces hyalurolyticus *were from Sigma-Aldrich. The restriction enzymes and T4 DNA ligase were from New England Biolabs Inc. Vespula venom extract used in western blots was a kind gift of Jørgen Nedergaard Larsen (ALK Abelló, Hørsholm, Denmark). Vespula venom extract used in histamine release assay was from ALK Abelló and contained 136 μg/ml protein.

### Strains and plasmids

The *E. coli *strains used for cloning were TOP10 and DH10B, both deficient in recombinase (*recA*) and endonuclease A (*endA*). The *Saccharomyces cerevisiae *strain was EBY100 (**a ***GAL1-AGA1*::*URA3 ura3-52 trp1 leu2*Δ*1 his3*Δ*200 pep4*::*HIS2 prb1*Δ*1.6R can1GAL*), Trp^-^Leu^-^, kindly provided by Dr. K. Dane Wittrup from MIT, USA. The plasmid pCTCON2 was also a gift from K. Dane Wittrup. Vector pPICZalphaA was purchased from Invitrogen. For longer storage the *E. coli *strains were stored in LB medium with 25% glycerol and yeast strains were stored in YPD with 15% glycerol at -80°C.

### Insects

Common wasps (*V. vulgaris*) were caught at two nests in Denmark. The wasps were classified as *V. vulgaris *based on their morphology: 15 mm-long body with characteristic yellow and black colors, a black anchor on the head, and black rings on the abdomen.

### RNA isolation

The venom sacs were removed with tweezers and immersed in RNAlater solution (Ambion, Inc.), stored overnight at 4°C and then transferred at -20°C for longer storage. For RNA isolation 10 venom sacs were removed from the RNAlater solution, grinded in liquid nitrogen, transferred to a FastPrep tube with 0.25-0.5 mm diameter acid-washed glass beads. 600 μl of RLT buffer was added and the samples were homogenized in a FastPrep FP120 machine (Qbiogene) at 2 cycles of 30 s with power set to 5.5. Further on the total RNA was extracted using RNeasy Mini kit (Qiagen) according to the manufacture's instructions. The final RNA concentration and purity was determined on spectrophotometer and on electrophoresis gel.

### Cloning of allergen genes

The genes encoding phospholipase A1 (Ves v 1), hyaluronidase (Ves v 2.0101) and antigen 5 (Ves v 5) were amplified from the total RNA of *V. vulgaris *using RT-PCR (Superscript III One-Step RT-PCR System with Platinum Taq DNA Polymerase, Invitrogen). The gene encoding hyaluronidase variant (Ves v 2.0201) could not be amplified. As a positive control for RT-PCR reaction we amplified fragments of 18 S and 28 S RNA. The primers used for RT-PCR reactions are shown in Table 2. Two genes, *ves v 1 *and *ves v 5*, were amplified together with their signal sequences. For the hyaluronidase (*ves v 2*) the signal sequence was not known, hence only the part encoding mature protein was amplified. 2.5 μl of the RT-PCR reaction were further used as a template in a 100-μl PCR reaction with Expand High Fidelity polymerase (Roche Diagnostics) and with the same primers to generate sufficient amounts of the fragment for cloning. The PCR reaction product was separated on 1.1% agarose gel stained with SYBR-safe (Invitrogen). The fragments of the correct size were excised with a scalpel and purified from the gel using GFX PCR DNA and gel band purification kit (GE Healthcare). The fragments were cloned into pCR^®^2.1-TOPO^® ^vector, using TOPO TA Cloning^® ^kit (Invitrogen) and transformed into chemically competent *E. coli *TOP10 cells. The transformants were selected on SOB plates with 20 mM MgCl_2_, 50 μg/ml kanamycin and 50 μg/ml X-gal. White colonies were screened for the presence of the cloned fragment using colony PCR with the fragment-specific primers (the same as used for RT-PCR). 3-4 correct clones were grown overnight and the plasmid was extracted using GeneElute™ Plasmid Miniprep kit (Sigma-Aldrich). After confirmation of the correct construct by restriction, the plasmids were sequenced at Eurofins MWG Operon or StarSEQ.

**Table 2:** List of primers

Primer name	Sequence	Application
vesv1_fw	ATGGAAGAAAATATGAATTTAAAG	Cloning genes from RNA
	
vesv1_rv	TTAAATTATCTTCCCCTTGTTATTG	
	
vesv2.0101_fw	TCCGAGAGACCGAAAAGAG	
	
vesv2.0101_rv	TTAGTTGACGGCTTCTGTCAC	
	
vesv2.0201_fw	GACAGAACAATTTGGCCTAAG	
	
vesv2.0201_rv	CTAAAAGTTTAACGGTGTGTTTTC	
	
vesv5_fw	ATGGAAATTAGTGGGCTC	
	
vesv5_rv	TTACTTTGTTTGATAAAGTTCCTC	
	
28SrRNAvesv_fw	AGCGTCAGCGGCGCTG	
	
28SrRNAvesv_rv	GAGACACTGACCGCGCTTG	

SD_vesv1_fw	*NheI* ATCA*GCTAGC*GGACCCAAATGTCCTTTTAATTC	Cloning genes into surface display vector
	
SD_vesv1_rv	*BamHI* CGAT*GGATCC*AATTATCTTCCCCTTGTTATTGC	
	
SD_vesv2_fw	*NheI* ATCA*GCTAGC*TCCGAGAGACCGAAAAG	
	
SD_vesv2_rv	*BamHI* CGAT*GGATCC*GTTGACGGCTTCTGTC	
	
SD_vesv5_fw	*NheI* ATCA*GCTAGC*AACAATTATTGTAAAATAAAATG	
	
SD_vesv5_rv	*BamHI* CGAT*GGATCC*CTTTGTTTGATAAAGTTC	

zeo_fw	*KpnI* TAGATT*GGTACC*CCCACACACCATAGCTTC	Cloning zeocin resistance gene
	
zeo_rv	*KpnI* GTCCTC*GGTACC*AGCTTGCAAATTAAAGC	

The first group of primers was used for amplification of four allergen genes and control 18 S rRNA from wasp venom sac RNA. The second group of primers was used for generating fragments with NheI and BamHI overhangs for cloning into surface display vector. The last set of primers was used for cloning zeocin resistance cassette with KpnI overhangs. The restriction sites are underlined.

### Construction of surface display vectors

The genes *ves v 1*, *ves v 2 *and *ves v 5 *were amplified from the corresponding TOPO plasmids TOPO_vesv1, TOPO_vesv2 and TOPO_vesv5 with forward and reverse primers indicated in Table 2 (primers starting with "SD") using Expand High Fidelity polymerase (Roche Diagnostics). The PCR products were separated on 1.1% agarose gel, cut and purified from the gels using GFX kit (GE Healthcare). The purified PCR fragments were restricted with NheI and BamHI enzymes and cloned into pCTCON2 vector, which was digested with the same restriction enzymes and gel purified. The ligation of the vector and the insert was performed with T4 DNA ligase (New England Biolabs, UK) at room temperature for 30 min. The ligation mixture was transformed into chemically competent *E. coli *DH10B cells and the transformants were selected on Luria-Bertani (LB) agar plates with 100 μg/ml ampicillin. The transformants carrying inserts were identified by colony PCR with the same primers as above. The correct transformants were grown in LB broth with 100 μg/ml ampicillin overnight and the plasmids were isolated using GenElute Miniprep kit (Sigma-Aldrich). The resulting surface display vectors were named pSD_vesv1, pSD_vesv2 and pSD_vesv5. The sequence of the plasmids was confirmed by restriction digestion and by sequencing.

### Construction of surface display vectors with zeocin resistance

The zeocin resistance cassette was PCR-amplified with zeo_fw and zeo_rv primers (Table 2) using plasmid pPICZalphaA as the template. The resulting fragment was purified from the gel, digested with KpnI and cloned into surface display vectors pSD_vesv2 and pSD_vesv5 cut with the same enzyme. The transformants were selected on LB plates with 100 μg/ml ampicillin and 25 μg/ml zeocin, giving pSD_vesv2_zeo and pSD_vesv5_zeo. For the construction of pSD_vesv1_zeo vector a different strategy was applied because the vesv1 gene contained a KpnI restriction site. The PCR-amplified and KpnI-digested zeocin resistance cassette was ligated with pCTCON2 vector digested with KpnI and the ligation mixture was transformed into *E. coli*. After selection of the zeocin-resistant clones, the plasmid pCTCON2_zeo was isolated and the vesv1 gene was cloned into it in the same way as it was cloned into pCTCON2 vector above.

### Yeast transformation

The surface display plasmids pCTCON2, pCTCON2_zeo, pSD_ves1, pSD_vesv2, pSD_vesv5, pSD_vesv1_zeo, pSD_vesv2_zeo, pSD_ves5_zeo were transformed into *S. cerevisiae *strain EBY100 as following. A single colony of *S. cerevisiae *was inoculated in 2 ml YPD medium overnight at 30°C with shaking. Next morning 50 ml YPD in a 500-ml shake flask was inoculated with the overnight culture to the start optical density (OD_600_) of 0.2 and grown until OD_600 _of 1.4-1.6. The cells were collected by centrifugation at 3,000 × g for 5 min, washed twice with 50 ml MilliQ water and once with 10 ml transformation buffer (20 mM HEPES in 1 M sorbitol). The cells were eventually resuspended in 100 μl transformation buffer. 40 μl of the cell suspension were mixed with 5 μl of plasmid preparation, electroporated on Gene Pulser (Bio-Rad Laboratories) at 1.5 kV. 1 ml transformation buffer was added to the cells immediately after electroporation and the cell suspension was transferred to a 15 ml tube. After incubation at 30°C for 1 hour the cells were centrifuged, resuspended in 100 μl buffer and volumes of 10 and 50 μl were plated on tryptophan-deficient SDCAA plates [36]. When transforming vectors with zeocin resistance cassettes the SDCAA was supplemented with 100 μg/ml zeocin. Colonies appeared after 2-3 days of incubation at 30°C. The presence of the vector was confirmed by colony-PCR using gene-specific primers.

### Cultivation and induction of yeast cells

The cultivation medium was SDCAA and SGCAA as described in Chao *et al. *[36], except that the phosphate concentration was reduced to 50 mM in order to avoid zeocin inhibition by high salt concentration. The SDCAA medium contained 20 g/L dextrose as carbon source, while in SGCAA medium the dextrose was replaced by the same amount of galactose. A single colony was inoculated in 20 ml SDCAA medium in 500-ml baffled flask and grown overnight at 30°C with shaking at 150 rpm to an OD_600 _of about 1-2. The cells were harvested by centrifugation and resuspended in SGCAA medium to OD_600 _of 1. The cultures were transferred into 500-ml baffled shake flasks and grown at room temperature (22-23°C) for 30 hours. The cultures were stored at +4°C until analysis, but never longer than one week.

### Growth experiment

For the growth rate experiments the cells were grown in SDCAA as above and then resuspended to the OD_600 _of 0.1 in SGCAA medium with or without 100 μg/ml zeocin. The cell suspensions were pipetted into a sterile 96-well flat-bottom microtiter plate (100 μl suspension per well) (Nunc 96-microwell plate from Thermo Scientific). Sterile SGCAA media was used as the background reference. All the samples were run in triplicates. The plates were covered by air-permeable transparent film and shaken during overnight incubation at room temperature. The OD_600 _readings were performed automatically every half an hour with Multiscan Accent (Thermo Scientific). The data was exported into Excel (Microsoft) and used to calculate the maximum specific growth rates during the exponential growth phase.

### SDS-Polyacrylamide Gel Electrophoresis

The gel was a pre-cast 10% NuPAGE^® ^Novex^® ^Bis-Tris electrophoresis gel from Invitrogen. The protein size marker was unstained or pre-stained PageRuler™ protein ladder from Fermentas (Germany). The samples were mixed with NuPAGE^® ^LDS sample buffer (4×) and NuPAGE^® ^reducing agent (10×) and heated at 70°C for 10 min before loading on the gel. The electrophoresis was performed at a constant voltage of 200 V for 35 min. The gel was used for western blot or stained with silver stain (Fermentas, Germany).

### Western blot

For western blot the proteins were elecrophoretically transferred onto 0.2 μm PVDF membrane Amersham Hybond™-P (GE Healthcare) using Mini Trans-Blot^® ^electrophoretic transfer cell (Bio-Rad Laboratories) at 100 V for 1 hour. The transfer was done in 25 mM Tris, 192 mM glycine, and 20% v/v ethanol buffer.

### Detection of expression with anti-C-myc antibody

The cells grown in SGCAA were analyzed for expression of the allergen using Alexa488-conjugated anti-C-myc antibody. The analysis was possible because the allergens were cloned in-frame with c-myc epitope present in the pCTCON2 vector. The C-myc epitope was positioned at the C-terminal of the proteins. The cell volume containing about 10^6 ^yeast cells (OD_600 _of 1 corresponds to about 10^7 ^cells/ml) was moved to an ependoff tube with 1 ml blocking buffer (5 g/L skim milk powder, 50 mM Tris, 150 mM sodium chloride, 0.1 g/L Tween 20, pH 7.5). The content was mixed by inversion and centrifuged at 4,000 × g for 2 min. The supernatant was removed with a pipette and the cells were resuspended in 45 μl blocking buffer and 5 μl anti-C-myc-Alexa488 and incubated in the dark at room temperature for 1 hour with occasional mixing. Non-labeled controls with omission of antibody were prepared as well. The cells were washed twice with cold TBST buffer (50 mM Tris, 150 mM sodium chloride, 0.1 g/L Tween 20, pH 7.5) and resuspended in 500 μl TBST. The cells were analyzed by flow cytometry on Cytomics FC 500 MPL flow cytometry system with MXP software (Beckman Coulter). The cell debris and other small particles were eliminated from analysis by setting the forward scatter threshold. Normally about 95-97% of the cells were gated.

### Binding of IgE from patient serum

The induced cells were analyzed for binding IgE from patients' sera. The cell volume containing about 10^6 ^yeast cells was mixed with PBSF buffer (8 g/L NaCl, 0.2 g/L KCl, 1.44 g/L Na_2_HPO_4_, 0.24 g/L KH_2_PO_4_, 1 g/L bovine serum albumin, pH 7.4) to the total volume of 1 ml. The cells were centrifuged at 4,000 × g for 2 min and washed with 1 ml PBSF buffer. The cells were pelleted again and resuspended in 50 μl PBSF. 12.5 μl of cell suspension was mixed with 12.5 μl of positive or negative sera pool and incubated at room temperature for 1.5 hours with occasional mixing. The cells were washed with 1 ml ice-cold PBSF twice and mixed with biotinylated anti-IgE antibody diluted 1:200 in PBSF (total volume of 80 μl). For the double labeling the anti-C-myc antibody was added together with anti-IgE antibody at the dilution 1:10. The mix was incubated at 4°C for 1 hour. The cells were centrifuged and washed twice with ice-cold PBSF and mixed with streptavidin-phycoerythrin diluted 1:1000 in PBSF (total volume of 100 μl). The mix was incubated at 4°C for 30 min, the cells were washed twice with ice-cold PBSF, resuspended in 400 μl PBSF and analyzed by flow cytometry as above.

### Histamine release assay

Peripheral blood mononuclear cells (PBMCs) from buffy coat blood (n = 3, non-allergic donor, anti-IgE responsive cells) were isolated by Lymphoprep gradient centrifugation. IgE was stripped off the basophils by a rebound in pH from 7.4 to 3.55 and back to 7.4. The PBMCs were then incubated 1 hour with wasp patient or control pool (non-allergic) serum to re-sensitize the basophils, which subsequently were mixed with erythrocytes and challenged in glass fiber coated microtiter plates with control yeast expressing CD20 or yeast expressing phospholipase A1 (Ves v 1), hyaluronidase (Ves v 2) or antigen 5 (Ves v 5). Released histamine bound to the glass fibers was coupled to o-phtahaldialdehyde, stabilized by HCLO_4_, and measured fluorometrically as described previously [37]. Results were expressed as percentage of total cellular histamine content and were considered positive when > 10%.

### Enzymatic assays

The enzymatic activity of hyaluronidase was measured by turbidimetric method of Dorfman [38]. The volumes were reduced for microtiter plate format. After incubation with hyaluronic acid the mixture was centrifuged to remove the cells and only supernatant was added to the acid albumin solution. Hyaluronidase from *S. hyalurolyticus *was used as a standard.

Phospholipase A1 enzymatic assay was performed using EnzChek^® ^phospholipase A1 assay kit from Invitrogen according to the manufacturer's protocol. The controls without reagents addition were performed to ensure that the yeast cells did not fluoresce at the used wavelength. The Lecitase^® ^Ultra (Invitrogen) was used as a standard.

The non-transformed yeast cells and the yeast cells expressing CD20 protein were used as the negative controls in both assays.

## Abbreviations

**FACS**: fluorescence activated cell sorting, a.k.a. flow cytometry; **c-myc tag**: a 10-amino acids long polypeptide protein tag with sequence EQKLISEEDL; **CCDs**: cross-reactive carbohydrate determinants; **AGA1 and AGA2**: two subunits of a-agglutinin complex in *Saccharomyces cerevisiae*; **GPI anchor**: glycosylphosphatidylinositol anchor; **Ves v 1**: phospholipase A1; allergen from *Vespula vulgaris *venom; **Ves v 2.0101**: hyaluronidase; allergen from *Vespula vulgaris *venom; **Ves v 2.0201**: allergen from *Vespula vulgaris *venom with similarity to Ves v 2.0101; function - unknown; **Ves v 5**: antigen 5; allergen from *Vespula vulgaris *venom; function unknown; **CD20**: B-lymphocyte antigen CD20; a non-glycosylated phosphoprotein expressed on the surface of all mature B-cells.

## Competing interests

The authors declare that they have no competing interests.

## Authors' contributions

IB initiated and performed the experimental study and drafted the manuscript. BJ participated in design of the study, carried out histamine release assays and revised the manuscript. IBS and LKP participated in the design and coordination of the study and revised the manuscript. All authors read and approved the final manuscript.
